# Supplementary material for: The anterior cingulate cortex and its role in controlling contextual fear memory to predatory threats
Source: eLife. 2022 Jan 5;11:e67007. doi: 10.7554/eLife.67007 (PMC8730726; doi:10.7554/eLife.67007)
Supplement: Figure 1—source data 1. [file elife-67007-fig1-data1.docx]

**De Lima et al. Figure 1 – Raw data**

**Behavioral data**

| **Animal** | **Phase** | **GROUP** | ***PET_Freez*** | ***PET_RA*** | ***PET_Exp*** | ***CONT_RA*** | ***CONT_Exp*** |
| --- | --- | --- | --- | --- | --- | --- | --- |
| C1 | Acquisition | **hM4D+** | 83,944 | 109,536 | 32,8184 | 53,86 | 144,66 |
| C5 | Acquisition | **hM4D+** | 92,128 | 108,872 | 33,376 | 47,78 | 172,16 |
| C10 | Acquisition | **hM4D+** | 94,192 | 107,552 | 28,336 | 65,04 | 132,42 |
| C16 | Acquisition | **hM4D+** | 88,208 | 89,728 | 41,24 | 61,64 | 136,5 |
| C17 | Acquisition | **hM4D+** | 107,432 | 95,224 | 29,28 | 37,42 | 136,16 |
| C24 | Acquisition | **hM4D+** | 81,896 | 106,384 | 34,704 | 50,84 | 140,32 |
| C25 | Acquisition | **hM4D+** | 101,368 | 97,344 | 28,56 | 66,28 | 141 |
| C11 | Acquisition | **hM4D-** | 82,136 | 100,392 | 41,944 | 163,28 | 44,46 |
| C12 | Acquisition | **hM4D-** | 92,208 | 91,568 | 28,568 | 130,46 | 62,62 |
| C20 | Acquisition | **hM4D-** | 91,16 | 111,552 | 36,192 | 118,18 | 65,44 |
| C21 | Acquisition | **hM4D-** | 115,544 | 94,744 | 22,568 | 145,7 | 44,64 |
| C22 | Acquisition | **hM4D-** | 117,472 | 97,088 | 24,248 | 152,24 | 48,44 |
| C23 | Acquisition | **hM4D-** | 85,584 | 104,736 | 26,248 | 131,42 | 59,44 |
| C3 | Expression | **hM4D+** | 86,944 | 122,712 | 23,448 | 48,62 | 149,68 |
| C6 | Expression | **hM4D+** | 82,504 | 114,152 | 37,936 | 68,26 | 112,88 |
| C9 | Expression | **hM4D+** | 71,376 | 124,68 | 33,04 | 54,22 | 161,8 |
| C13 | Expression | **hM4D+** | 99,376 | 98,512 | 17,04 | 42,18 | 153,9 |
| C14 | Expression | **hM4D+** | 78,832 | 107,032 | 24,496 | 45,4 | 150,18 |
| C15 | Expression | **hM4D+** | 93,416 | 91 | 16,48 | 65,08 | 112,24 |
| C26 | Expression | **hM4D+** | 96,04 | 92,616 | 32,168 | 43,4 | 127,14 |
| C19 | Expression | **hM4D-** | 105,08 | 92,448 | 22,256 | 161,46 | 30,44 |
| C28 | Expression | **hM4D-** | 95,72 | 99,496 | 36,376 | 138,5 | 50,02 |
| C1 | Expression | **hM4D-** | 84,288 | 119,848 | 31,048 | 151,74 | 58,84 |
| C2 | Expression | **hM4D-** | 89,176 | 120,08 | 26,072 | 125,7 | 56,04 |
| C4 | Expression | **hM4D-** | 94,632 | 105,76 | 30,88 | 133,04 | 56,62 |
