# Supplementary material for: The anterior cingulate cortex and its role in controlling contextual fear memory to predatory threats
Source: eLife. 2022 Jan 5;11:e67007. doi: 10.7554/eLife.67007 (PMC8730726; doi:10.7554/eLife.67007)
Supplement: Figure 2—source data 1. [file elife-67007-fig2-data1.docx]

**De Lima et al. Figure 2 – Raw data**

| **ORBm PET** | Total Fos | Total FG | Fos/FG |  | **CL PET** | Total Fos | Total FG | Fos/FG |
| --- | --- | --- | --- | --- | --- | --- | --- | --- |
| c1 | 183 | 105 | 40 |  | c1 | 80 | 88 | 47 |
| c2 | 150 | 103 | 41 |  | c2 | 74 | 77 | 45 |
| **c3** | 215 | 173 | 48 |  | c3 | 53 | 52 | 27 |
| **c4** | 214 | 168 | 51 |  | c4 | 51 | 53 | 28 |
| c5 | 160 | 155 | 47 |  | c5 | 52 | 65 | 32 |
| c6 | 162 | 141 | 38 |  | c6 | 61 | 59 | 35 |
|  |  |  |  |  |  |  |  |  |
| **PL PET** | Total Fos | Total FG | Fos/FG |  | **BLA PET** | Total Fos | Total FG | Fos/FG |
| c1 | 197 | 186 | 54 |  | c1 | 76 | 61 | 16 |
| c2 | 188 | 179 | 65 |  | c2 | 86 | 60 | 16 |
| c3 | 117 | 174 | 44 |  | c3 | 77 | 68 | 13 |
| c4 | 123 | 173 | 48 |  | c4 | 87 | 69 | 18 |
| c5 | 136 | 159 | 40 |  | c5 | 81 | 66 | 17 |
| c6 | 148 | 155 | 48 |  | c6 | 75 | 63 | 11 |
|  |  |  |  |  |  |  |  |  |
| **CLA PET** | Total Fos | Total FG | Fos/FG |  | **VIS PET** | Total Fos | Total FG | Fos/FG |
| c1 | 74 | 85 | 19 |  | c1 | 220 | 143 | 76 |
| c2 | 66 | 84 | 18 |  | c2 | 226 | 131 | 68 |
| c3 | 68 | 79 | 21 |  | c3 | 202 | 140 | 71 |
| c4 | 72 | 68 | 23 |  | c4 | 184 | 115 | 56 |
| c5 | 93 | 106 | 32 |  | c5 | 126 | 112 | 51 |
| c6 | 88 | 96 | 35 |  | c6 | 124 | 113 | 52 |
|  |  |  |  |  |  |  |  |  |
| **AM PET** | Total Fos | Total FG | Fos/FG |  | **RSP PET** | Total Fos | Total FG | Fos/FG |
| c1 | 76 | 112 | 68 |  | **c1** | 183 | 160 | 83 |
| c2 | 70 | 115 | 56 |  | c2 | 157 | 150 | 79 |
| c3 | 72 | 102 | 53 |  | c3 | 213 | 167 | 87 |
| c4 | 83 | 131 | 65 |  | c4 | 170 | 147 | 72 |
| c5 | 73 | 108 | 54 |  | c5 | 161 | 138 | 66 |
| c6 | 98 | 142 | 73 |  | c6 | 154 | 142 | 74 |

| **ENTl PET** | Total Fos | Total FG | Fos/FG |  | **HIPv PET** | Total Fos | Total FG | Fos/FG |
| --- | --- | --- | --- | --- | --- | --- | --- | --- |
| c1 | 71 | 26 | 7 |  | c1 | 81 | 32 | 11 |
| c2 | 73 | 31 | 8 |  | c2 | 43 | 22 | 7 |
| c3 | 75 | 22 | 7 |  | **c3** | 94 | 40 | 10 |
| c4 | 76 | 31 | 9 |  | **c4** | 80 | 41 | 10 |
| c5 | 65 | 28 | 8 |  | **c5** | 98 | 47 | 13 |
| c6 | 74 | 36 | 9 |  | **c6** | 79 | 30 | 9 |

**De Lima et al. Figure 2 – Raw data**

|  |  |  |  |  | Confidence interval |  | Bar Sizes |  |  |
| --- | --- | --- | --- | --- | --- | --- | --- | --- | --- |
|  |  | **Fos / FG** | **Total FG** | **Proportion** | **LL** | **UL** | **Left** | **Right** | **MOEav** |
| 1 | **ORBm PET** | 265 | 845 | 0,314 | 0,283 | 0,346 | 0,030 | 0,032 | 0,031 |
| 2 | **PL PET** | 299 | 1026 | 0,291 | 0,264 | 0,320 | 0,027 | 0,029 | 0,028 |
| 3 | **CLA PET** | 148 | 518 | 0,286 | 0,248 | 0,326 | 0,037 | 0,040 | 0,039 |
| 4 | **AM PET** | 369 | 710 | 0,520 | 0,483 | 0,556 | 0,037 | 0,037 | 0,037 |
| 5 | **CL PET** | 214 | 394 | 0,543 | 0,494 | 0,592 | 0,049 | 0,049 | 0,049 |
| 6 | **BLA PET** | 91 | 387 | 0,235 | 0,196 | 0,280 | 0,040 | 0,045 | 0,042 |
| 7 | **VIS PET** | 374 | 754 | 0,496 | 0,460 | 0,532 | 0,036 | 0,036 | 0,036 |
| 8 | **RSP PET** | 461 | 904 | 0,510 | 0,477 | 0,542 | 0,033 | 0,032 | 0,033 |
| 9 | **ENTl PET** | 48 | 174 | 0,276 | 0,215 | 0,347 | 0,061 | 0,071 | 0,066 |
| 10 | **HIPv PET** | 60 | 212 | 0,283 | 0,227 | 0,347 | 0,056 | 0,064 | 0,060 |
