# Supplementary material for: The anterior cingulate cortex and its role in controlling contextual fear memory to predatory threats
Source: eLife. 2022 Jan 5;11:e67007. doi: 10.7554/eLife.67007 (PMC8730726; doi:10.7554/eLife.67007)
Supplement: Figure 3—source data 1. [file elife-67007-fig3-data1.docx]

**De Lima et al. Figure 3 – Raw data**

| **ORBm - Context** | Total Fos | Total FG | Fos/FG |  | **CL Context** | Total Fos | Total FG | Fos /FG |
| --- | --- | --- | --- | --- | --- | --- | --- | --- |
| c7 | 132 | 138 | 34 |  | c7 | 62 | 90 | 37 |
| c8 | 138 | 175 | 43 |  | c8 | 45 | 69 | 25 |
| c9 | 112 | 134 | 45 |  | c9 | 42 | 74 | 27 |
| c10 | 131 | 198 | 55 |  | c10 | 51 | 67 | 28 |
| c11 | 126 | 122 | 32 |  | c11 | 39 | 58 | 21 |
| c12 | 115 | 124 | 28 |  | c12 | 46 | 70 | 25 |
|  |  |  |  |  |  |  |  |  |
|  |  |  |  |  |  |  |  |  |
| **PL - Context** | Total Fos | Total FG | Fos/FG |  | **BLA - Context** | Total Fos | Total FG | Fos/ FG |
| c7 | 128 | 198 | 38 |  | c7 | 52 | 54 | 14 |
| c8 | 105 | 131 | 33 |  | c8 | 43 | 45 | 9 |
| c9 | 101 | 138 | 30 |  | c9 | 57 | 55 | 17 |
| c10 | 89 | 125 | 34 |  | c10 | 51 | 48 | 14 |
| c11 | 95 | 165 | 46 |  | c11 | 37 | 48 | 10 |
| c12 | 126 | 151 | 42 |  | c12 | 45 | 60 | 12 |
|  |  |  |  |  |  |  |  |  |
|  |  |  |  |  |  |  |  |  |
| **CLA - Context** | Total Fos | Total FG | Fos/FG |  | **VIS - Context** | Total Fos | Total FG | Fos /FG |
| c7 | 47 | 94 | 22 |  | c7 | 98 | 133 | 33 |
| c8 | 40 | 68 | 12 |  | c8 | 81 | 108 | 25 |
| c9 | 58 | 106 | 23 |  | c9 | 130 | 132 | 44 |
| c10 | 71 | 109 | 28 |  | c10 | 106 | 105 | 37 |
| c11 | 39 | 86 | 17 |  | c11 | 77 | 111 | 30 |
| c12 | 71 | 108 | 32 |  | c12 | 72 | 114 | 26 |
|  |  |  |  |  |  |  |  |  |
|  |  |  |  |  |  |  |  |  |
| **AM - Context** | Total Fos | Total FG | Fos /FG |  | **RSP - Context** | Total Fos | Total FG | Fos/FG |
| c7 | 41 | 109 | 26 |  | c7 | 120 | 134 | 44 |
| c8 | 39 | 109 | 24 |  | c8 | 86 | 116 | 33 |
| c9 | 58 | 131 | 37 |  | **c9** | 145 | 160 | 57 |
| c10 | 42 | 130 | 25 |  | c10 | 129 | 149 | 48 |
| c11 | 46 | 122 | 22 |  | c11 | 85 | 118 | 33 |
| c12 | 37 | 123 | 23 |  | c12 | 111 | 148 | 39 |
|  |  |  |  |  |  |  |  |  |
| **ENTl- Context** | **Total Fos** | **Total FG** | **Fos/FG** |  | **HIPv Context** | **Total Fos** | **Total FG** | **Fos + FG** |
| **c7** | **61** | **44** | **13** |  | **c7** | **36** | **49** | **6** |
| **c8** | **52** | **43** | **12** |  | **c8** | **56** | **46** | **7** |
| **c9** | **39** | **36** | **10** |  | **c9** | **31** | **29** | **3** |
| **c10** | **38** | **28** | **8** |  | **c10** | **29** | **34** | **3** |
| **c11** | **32** | **25** | **6** |  | **c11** | **37** | **41** | **5** |
| **c12** | **45** | **31** | **10** |  | **c12** | **34** | **40** | **6** |
|  |  |  |  |  |  |  |  |  |

**De Lima et al. Figure 3 – Raw data**

|  |  |  |  |  | Confidence interval |  | Bar Sizes |  |  |
| --- | --- | --- | --- | --- | --- | --- | --- | --- | --- |
|  |  | **Fos/FG** | **Total FG** | **Proportion** | **LL** | **UL** | **Left** | **Right** | **MOEav** |
| 1 | **ORBm CONT** | 237 | 891 | 0,266 | 0,238 | 0,296 | 0,028 | 0,030 | 0,029 |
| 2 | **PL CONT** | 223 | 908 | 0,246 | 0,219 | 0,275 | 0,027 | 0,029 | 0,028 |
| 3 | **CLA CONT** | 134 | 571 | 0,235 | 0,202 | 0,271 | 0,033 | 0,036 | 0,035 |
| 4 | **AM CONT** | 157 | 724 | 0,217 | 0,188 | 0,248 | 0,028 | 0,031 | 0,030 |
| 5 | **CL CONT** | 163 | 428 | 0,381 | 0,336 | 0,428 | 0,045 | 0,047 | 0,046 |
| 6 | **BLA CONT** | 76 | 310 | 0,245 | 0,201 | 0,296 | 0,045 | 0,051 | 0,048 |
| 7 | **VIS CONT** | 195 | 703 | 0,277 | 0,246 | 0,312 | 0,032 | 0,034 | 0,033 |
| 8 | **RSP CONT** | 254 | 825 | 0,308 | 0,277 | 0,340 | 0,031 | 0,032 | 0,031 |
| 9 | **ENTl CONT** | 59 | 207 | 0,285 | 0,228 | 0,350 | 0,057 | 0,065 | 0,061 |
| 10 | **HIPv CONT** | 30 | 239 | 0,126 | 0,089 | 0,174 | 0,036 | 0,048 | 0,042 |
