# Supplementary material for: The anterior cingulate cortex and its role in controlling contextual fear memory to predatory threats
Source: eLife. 2022 Jan 5;11:e67007. doi: 10.7554/eLife.67007 (PMC8730726; doi:10.7554/eLife.67007)
Supplement: Figure 4—source data 1. [file elife-67007-fig4-data1.docx]

**De Lima et al. Figure 4 – Raw data**

**Behavioral data**

| **Animal** | **GROUP** | ***PET_Freez*** | ***PET_RA*** | ***PET_Exp*** | ***Cont_RA*** | ***Cont_Exp*** |
| --- | --- | --- | --- | --- | --- | --- |
| C110 | **HR+** | 84,976 | 115,04 | 33,784 | 40,9 | 164,96 |
| C111 | **HR+** | 100,72 | 109,928 | 26,192 | 57,24 | 134,48 |
| C116 | **HR+** | 62,216 | 125,28 | 39,864 | 43,82 | 165,54 |
| C118 | **HR+** | 66,328 | 104,048 | 53,312 | 49,44 | 144,1 |
| C119 | **HR+** | 105,128 | 95,304 | 30,752 | 39 | 145,52 |
| C120 | **HR+** | 122,816 | 91,16 | 24,168 | 50,02 | 114,36 |
| C121 | **HR+** | 79,704 | 117,744 | 29,296 | 26,6 | 164,66 |
| C122 | **HR+** | 73,968 | 112,736 | 46,776 | 49,22 | 161,18 |
| C123 | **HR-** | 89,64 | 115,84 | 25,56 | 190,58 | 24,98 |
| C124 | **HR-** | 120,288 | 97,672 | 17,928 | 168,3 | 36,8 |
| C125 | **HR-** | 124,44 | 91,328 | 22,264 | 162,74 | 32,82 |
| C126 | **HR-** | 82,704 | 120,288 | 34,008 | 176,36 | 38,72 |
| C127 | **HR-** | 85,072 | 111,696 | 42,304 | 134,22 | 55,26 |
| C128 | **HR-** | 82,76 | 108,448 | 42,288 | 170,94 | 47,82 |
| C129 | **HR-** | 96,472 | 102,8 | 34,824 | 146,88 | 38,26 |
