# Supplementary material for: The anterior cingulate cortex and its role in controlling contextual fear memory to predatory threats
Source: eLife. 2022 Jan 5;11:e67007. doi: 10.7554/eLife.67007 (PMC8730726; doi:10.7554/eLife.67007)
Supplement: Figure 5—source data 1. — Cell counting – spreadsheet of raw values for panels (E) and (G). [file elife-67007-fig5-data1.docx]

**De Lima et al. Figure 5 – Raw data**

**Behavioral data**

| **Animal** | **Phase** | **GROUP** | ***PET_Freez*** | ***PET_ RA*** | ***PET_Exp*** | ***CONT_RA*** | ***CONT_Exp*** |
| --- | --- | --- | --- | --- | --- | --- | --- |
| C91 | Acquisition | **HR+** | 86,448 | 94,864 | 38,768 | 46,44 | 143,12 |
| C95 | Acquisition | **HR+** | 109,448 | 93,28 | 17,712 | 68,06 | 132,62 |
| C96 | Acquisition | **HR+** | 103,592 | 110,16 | 25,616 | 40,44 | 156,68 |
| C97 | Acquisition | **HR+** | 88,632 | 112,984 | 37,384 | 39,82 | 126,78 |
| C98 | Acquisition | **HR+** | 102,984 | 114,192 | 22,656 | 57,42 | 142,94 |
| C99 | Acquisition | **HR+** | 104,104 | 108,088 | 17,768 | 46 | 158,54 |
| C104 | Acquisition | **HR+** | 89,832 | 100,344 | 14,76 | 64,86 | 124,98 |
| C108 | Acquisition | **HR+** | 96,816 | 118,056 | 15,512 | 36,68 | 155,8 |
| C1 | Acquisition | **HR-** | 90,288 | 113,24 | 34,448 | 196,26 | 30,82 |
| C2 | Acquisition | **HR-** | 135,48 | 81,936 | 18,512 | 187,96 | 45,02 |
| C3 | Acquisition | **HR-** | 109,704 | 85,632 | 18,12 | 145,2 | 39,46 |
| C4 | Acquisition | **HR-** | 97,088 | 106,568 | 35,424 | 180,82 | 50,36 |
| C5 | Acquisition | **HR-** | 88,952 | 118,248 | 29,824 | 182,48 | 29,58 |
| C6 | Acquisition | **HR-** | 114,296 | 102,952 | 20,096 | 166,944 | 34,008 |
| C100 | Expression | **HR+** | 89,648 | 113,896 | 19,688 | 113,68 | 67,84 |
| C101 | Expression | **HR+** | 89,312 | 110,576 | 23,488 | 126,54 | 47,86 |
| C102 | Expression | **HR+** | 78,944 | 107,76 | 35,808 | 147,76 | 53,02 |
| C103 | Expression | **HR+** | 81,848 | 111,048 | 27,752 | 152,22 | 38,58 |
| C107 | Expression | **HR+** | 115,04 | 85,432 | 14,664 | 163,36 | 44,62 |
| C109 | Expression | **HR+** | 82,344 | 109,856 | 33,912 | 151,22 | 34,2 |
| C110 | Expression | **HR+** | 108,664 | 90,144 | 25,344 | 190,56 | 34,42 |
| C111 | Expression | **HR+** | 106,008 | 106,648 | 23,152 | 124,14 | 65,64 |
| C130 | Expression | **HR-** | 88,616 | 116,664 | 18,512 | 150,94 | 44,2 |
| C131 | Expression | **HR-** | 100,648 | 109,832 | 21,48 | 143,18 | 45,656 |
| C132 | Expression | **HR-** | 137,56 | 81,384 | 18,68 | 161,14 | 48,54 |
| C133 | Expression | **HR-** | 95,88 | 98,192 | 31,568 | 167,94 | 48,6 |
| C134 | Expression | **HR-** | 95,112 | 96,48 | 37,728 | 137,6 | 56,24 |
| C135 | Expression | **HR-** | 110,728 | 103,696 | 23,544 | 139,656 | 46,712 |

**De Lima et al. Figure 5 – Raw data**

**Cell counting data**

| **BLA-PET** | **Total FG** | **FG-Fos** | **Total DAPI** | **Total FOS** |
| --- | --- | --- | --- | --- |
| C200 | 27 | 22 | 640 | 355 |
| C200 | 31 | 24 | 981 | 499 |
| C200 | 29 | 25 | 650 | 313 |
| C201 | 57 | 46 | 1146 | 464 |
| C201 | 42 | 38 | 625 | 246 |
| C201 | 63 | 52 | 1435 | 602 |
| C202 | 68 | 55 | 1086 | 470 |
| C202 | 72 | 60 | 1077 | 528 |
| C202 | 45 | 37 | 652 | 297 |
| C203 | 63 | 49 | 996 | 388 |
| C203 | 61 | 53 | 898 | 315 |
| C203 | 61 | 54 | 1046 | 354 |
| **TOTAL** | **619** | **515** | **11232** | **4831** |

| **BLA - Context** | **Total FG** | **FG-Fos** | **Total DAPI** | **Total FOS** |
| --- | --- | --- | --- | --- |
| C204 | 29 | 6 | 670 | 220 |
| C204 | 24 | 5 | 570 | 187 |
| C204 | 19 | 3 | 421 | 116 |
| C205 | 19 | 6 | 453 | 127 |
| C205 | 20 | 3 | 482 | 144 |
| C205 | 32 | 8 | 614 | 156 |
| C206 | 40 | 12 | 583 | 128 |
| C206 | 39 | 8 | 627 | 174 |
| C206 | 29 | 6 | 471 | 152 |
| C207 | 22 | 6 | 465 | 137 |
| C207 | 24 | 9 | 514 | 175 |
| C207 | 28 | 6 | 421 | 143 |
| **TOTAL** | **325** | **78** | **6291** | **1859** |
