# Supplementary material for: The anterior cingulate cortex and its role in controlling contextual fear memory to predatory threats
Source: eLife. 2022 Jan 5;11:e67007. doi: 10.7554/eLife.67007 (PMC8730726; doi:10.7554/eLife.67007)
Supplement: Figure 6—source data 1. — Cell counting – spreadsheet of raw values for panels (E) and (G). [file elife-67007-fig6-data1.docx]

**De Lima et al. Figure 6 – Raw data**

**Behavioral data**

| **Animal** | **Phase** | **GROUP** | **PET_Freez** | **PET_RA** | **PET_Exp** | **CONT_RA** | **CONT_Exp** |
| --- | --- | --- | --- | --- | --- | --- | --- |
| C30 | Acquisition | HR+ | 93,568 | 115,024 | 31,048 | 33,2 | 161,18 |
| C33 | Acquisition | HR+ | 102,688 | 102,32 | 32,904 | 61,04 | 157,48 |
| C34 | Acquisition | HR+ | 93,344 | 99,784 | 27,536 | 43,08 | 186,42 |
| C35 | Acquisition | HR+ | 85,384 | 118 | 26,816 | 32,8 | 176,82 |
| C36 | Acquisition | HR+ | 98,776 | 106,312 | 20,808 | 39,816 | 187,92 |
| C40 | Acquisition | HR+ | 98,472 | 111,128 | 17,568 | 41,26 | 158,44 |
| C11 | Acquisition | HR- | 90,136 | 103,592 | 38,744 | 157,28 | 50,46 |
| C12 | Acquisition | HR- | 91,808 | 102,768 | 30,968 | 150,46 | 60,62 |
| C20 | Acquisition | HR- | 101,56 | 109,952 | 24,992 | 144,18 | 69,44 |
| C23 | Acquisition | HR- | 82,384 | 98,336 | 31,048 | 111,42 | 53,44 |
| C61 | Acquisition | HR- | 83,336 | 121,144 | 33,216 | 146,24 | 47,8 |
| C49 | Expression | HR+ | 84,832 | 110,776 | 27,688 | 145,32 | 48,82 |
| C83 | Expression | HR+ | 90,672 | 115,544 | 31,648 | 120,26 | 50,24 |
| C84 | Expression | HR+ | 86,152 | 116,008 | 28,896 | 119,08 | 56,62 |
| C86 | Expression | HR+ | 113,248 | 92,616 | 31,848 | 146,34 | 48,16 |
| C88 | Expression | HR+ | 98,408 | 102,72 | 35,232 | 168,14 | 37,38 |
| C89 | Expression | HR+ | 106,912 | 88,232 | 26,664 | 173,28 | 30,64 |
| C145 | Expression | HR- | 91,168 | 108,656 | 34,432 | 130,7 | 57,44 |
| C146 | Expression | HR- | 97,152 | 102,376 | 36,672 | 147,54 | 44,6 |
| C147 | Expression | HR- | 121,56 | 90,96 | 26,808 | 157,94 | 33,4 |
| C148 | Expression | HR- | 118,872 | 77,008 | 23,84 | 164,74 | 47,24 |
| C149 | Expression | HR- | 104,168 | 111,136 | 24,512 | 137,64 | 51,8 |
|  |  |  |  |  |  |  |  |

**De Lima et al. Figure 6 – Raw data**

**Cell counting data**

| **PERI - PET** | **Total FG** | **FG-Fos** | **Total DAPI** | **Total FOS** |
| --- | --- | --- | --- | --- |
| C216 | 36 | 20 | 860 | 337 |
| C216 | 33 | 17 | 761 | 331 |
| C216 | 28 | 14 | 525 | 225 |
| C217 | 46 | 27 | 886 | 298 |
| C217 | 36 | 22 | 880 | 480 |
| C217 | 30 | 12 | 722 | 328 |
| C218 | 36 | 14 | 852 | 364 |
| C218 | 35 | 14 | 829 | 349 |
| C218 | 18 | 8 | 411 | 186 |
| C219 | 15 | 6 | 441 | 141 |
| C219 | 17 | 8 | 522 | 285 |
| C219 | 27 | 11 | 633 | 267 |
| **TOTAL** | **357** | **173** | **8322** | **3591** |

| **PERI - Context** | **Total FG** | **FG-Fos** | **Total DAPI** | **Total FOS** |
| --- | --- | --- | --- | --- |
| C220 | 44 | 6 | 788 | 220 |
| C220 | 52 | 9 | 984 | 181 |
| C220 | 53 | 8 | 917 | 195 |
| C221 | 34 | 4 | 767 | 229 |
| C221 | 31 | 4 | 782 | 198 |
| C221 | 30 | 5 | 721 | 137 |
| C222 | 29 | 4 | 756 | 141 |
| C222 | 24 | 4 | 466 | 145 |
| C222 | 25 | 5 | 537 | 178 |
| C223 | 59 | 9 | 1122 | 228 |
| C223 | 59 | 10 | 993 | 213 |
| C223 | 33 | 4 | 770 | 190 |
| **TOTAL** | **473** | **72** | **9603** | **2255** |
