# Supplementary material for: The anterior cingulate cortex and its role in controlling contextual fear memory to predatory threats
Source: eLife. 2022 Jan 5;11:e67007. doi: 10.7554/eLife.67007 (PMC8730726; doi:10.7554/eLife.67007)
Supplement: Figure 7—source data 1. — Cell counting – spreadsheet of raw values for panels (E) and (G). [file elife-67007-fig7-data1.docx]

**De Lima et al. Figure 7 – Raw data**

**Behavioral data**

| **Animal** | **Phase** | **GROUP** | ***PET_Freez*** | ***PET_RA*** | ***PET_Exp*** | ***CONT_RA*** | ***CONT_RA*** |
| --- | --- | --- | --- | --- | --- | --- | --- |
| C31 | Acquisition | **HR+** | 108,888 | 101,952 | 22,976 | 156,72 | 26,36 |
| C55 | Acquisition | **HR+** | 93,632 | 109,992 | 35,464 | 195,96 | 21,64 |
| C56 | Acquisition | **HR+** | 92,352 | 109,96 | 26,344 | 112,1 | 38,24 |
| C60 | Acquisition | **HR+** | 89,552 | 116,112 | 33,856 | 151,78 | 28,02 |
| C41 | Acquisition | **HR+** | 109,8 | 85,888 | 36,512 | 182,78 | 32,2 |
| C42 | Acquisition | **HR+** | 96,6 | 118,264 | 20,416 | 174,96 | 30,82 |
| C7 | Acquisition | **HR-** | 96,2 | 97,232 | 36,984 | 137,86 | 35,64 |
| C8 | Acquisition | **HR-** | 106,048 | 98,136 | 28,592 | 165,62 | 43,56 |
| C9 | Acquisition | **HR-** | 92,904 | 105,68 | 38,928 | 122,52 | 41,04 |
| C10 | Acquisition | **HR-** | 101,488 | 89,976 | 28,12 | 151,06 | 25,92 |
| C11 | Acquisition | **HR-** | 92,952 | 111,136 | 33,424 | 187,02 | 36,46 |
| C57 | Expression | **HR+** | 82,864 | 122,216 | 33,48 | 99,68 | 49 |
| C58 | Expression | **HR+** | 114,4 | 101,7592 | 19,288 | 183,6 | 25,4 |
| C81 | Expression | **HR+** | 112,656 | 99,688 | 21,608 | 130,04 | 34,22 |
| C82 | Expression | **HR+** | 100,592 | 120,84 | 17,608 | 153,94 | 38,22 |
| C87 | Expression | **HR+** | 119,464 | 94,816 | 17,048 | 131,88 | 39,38 |
| C90 | Expression | **HR+** | 92,944 | 99,328 | 36,432 | 121,2 | 45,62 |
| C93 | Expression | **HR+** | 97,248 | 109,984 | 31,216 | 148,6 | 37,64 |
| C140 | Expression | **HR-** | 95,712 | 101,408 | 38,312 | 134,5 | 42,02 |
| C141 | Expression | **HR-** | 101,56 | 108,056 | 29,24 | 124,28 | 41,82 |
| C142 | Expression | **HR-** | 70,456 | 123,4 | 29,376 | 101,42 | 37,92 |
| C143 | Expression | **HR-** | 110,416 | 109,44 | 18,8 | 180,58 | 26,8 |
| C144 | Expression | **HR-** | 89,232 | 113,544 | 33,792 | 140,1 | 48,44 |

**De Lima et al. Figure 7 – Raw data**

**Cell counting data**

| **POST - PET** | **Total FG** | **FG-Fos** | **Total DAPI** | **Total FOS** |
| --- | --- | --- | --- | --- |
| C224 | 11 | 2 | 688 | 295 |
| C224 | 17 | 3 | 1002 | 402 |
| C224 | 8 | 1 | 502 | 220 |
| C225 | 7 | 2 | 519 | 201 |
| C225 | 15 | 4 | 705 | 282 |
| C225 | 10 | 2 | 475 | 226 |
| C226 | 8 | 2 | 460 | 185 |
| C226 | 9 | 2 | 552 | 225 |
| C226 | 5 | 1 | 231 | 115 |
| C227 | 22 | 5 | 1038 | 422 |
| C227 | 5 | 1 | 185 | 89 |
| C227 | 9 | 2 | 580 | 238 |
| **TOTAL** | **126** | **27** | **6937** | **2900** |

| **POST - Context** | **Total FG** | **FG-Fos** | **Total DAPI** | **Total FOS** |
| --- | --- | --- | --- | --- |
| C228 | 6 | 1 | 322 | 90 |
| C228 | 14 | 3 | 705 | 175 |
| C228 | 11 | 2 | 637 | 124 |
| C229 | 5 | 1 | 312 | 88 |
| C229 | 8 | 2 | 658 | 216 |
| C229 | 16 | 4 | 755 | 194 |
| C230 | 16 | 3 | 906 | 201 |
| C230 | 8 | 1 | 559 | 137 |
| C230 | 5 | 1 | 350 | 82 |
| C231 | 13 | 2 | 597 | 176 |
| C231 | 18 | 3 | 852 | 186 |
| C231 | 16 | 3 | 855 | 192 |
| **TOTAL** | **136** | **26** | **7508** | **1861** |
