# Supplementary material for: The anterior cingulate cortex and its role in controlling contextual fear memory to predatory threats
Source: eLife. 2022 Jan 5;11:e67007. doi: 10.7554/eLife.67007 (PMC8730726; doi:10.7554/eLife.67007)
Supplement: Figure 8—source data 1. — Cell counting – spreadsheet of raw values of panels (E) and (G). [file elife-67007-fig8-data1.docx]

**De Lima et al. Figure 8 – Raw data**

**Behavioral data**

| **Animal** | **Phase** | **GROUP** | **PET_Freez** | **PET_RA** | **PET_Exp** | **CONT_RA** | **CONT_Exp** |
| --- | --- | --- | --- | --- | --- | --- | --- |
| C50 | Acquisition | HR+ | 99,472 | 102,424 | 34,664 | 135,48 | 39,1 |
| C51 | Acquisition | HR+ | 81,568 | 109,856 | 33,128 | 196,12 | 18,8 |
| C54 | Acquisition | HR+ | 88,656 | 117,184 | 33,784 | 181,46 | 34,34 |
| C59 | Acquisition | HR+ | 96,56 | 107,464 | 28,088 | 188,58 | 18,2 |
| C75 | Acquisition | HR+ | 109,536 | 91,528 | 30,184 | 126,5 | 55,86 |
| C76 | Acquisition | HR+ | 98,584 | 112,904 | 20,408 | 151,94 | 48,44 |
| C77 | Acquisition | HR+ | 115,832 | 107,528 | 13,688 | 156,24 | 30,42 |
| C161 | Acquisition | HR- | 88,056 | 115,632 | 36,032 | 175,2 | 32,42 |
| C162 | Acquisition | HR- | 117,672 | 95,104 | 19,92 | 191,2 | 43,82 |
| C163 | Acquisition | HR- | 128,76 | 88,576 | 20,888 | 143,92 | 43,6 |
| C164 | Acquisition | HR- | 94,88 | 104,44 | 30,576 | 179,92 | 47,62 |
| C165 | Acquisition | HR- | 92,656 | 116,52 | 30,112 | 186,36 | 21,8 |
| C52 | Expression | HR+ | 86,584 | 114,256 | 27,472 | 31,84 | 145,02 |
| C53 | Expression | HR+ | 93,696 | 119,856 | 26,184 | 68,84 | 112,7 |
| C70 | Expression | HR+ | 120,776 | 85,072 | 33,616 | 67,88 | 114,84 |
| C71 | Expression | HR+ | 98,048 | 113,16 | 23,84 | 43,4 | 141,5 |
| C72 | Expression | HR+ | 106,552 | 108,808 | 23,616 | 42,82 | 155,08 |
| C73 | Expression | HR+ | 95,488 | 102,984 | 28,704 | 57,04 | 136,1 |
| C74 | Expression | HR+ | 92,928 | 114,504 | 30,608 | 42,64 | 127,26 |
| C79 | Expression | HR+ | 111,448 | 113,808 | 14,168 | 65,92 | 157,94 |
| C85 | Expression | HR+ | 126,408 | 90,44 | 21,76 | 47,44 | 127,92 |
| C160 | Expression | HR- | 107,336 | 101,096 | 20,32 | 139,28 | 30,82 |
| C166 | Expression | HR- | 108,08 | 113,288 | 17,848 | 189,02 | 20,62 |
| C167 | Expression | HR- | 114,136 | 101,152 | 20,576 | 181,42 | 25,8 |
| C168 | Expression | HR- | 111,424 | 98,712 | 29,464 | 179,38 | 37,6 |
| C169 | Expression | HR- | 108,288 | 101,776 | 29,368 | 161,72 | 42,04 |
|  |  |  |  |  |  |  |  |

**De Lima et al. Figure 8 – Raw data**

**Cell counting data**

| **PAG - PET** | **Total FG** | **FG-Fos** | **Total DAPI** | **Total FOS** |
| --- | --- | --- | --- | --- |
| C208 | 25 | 5 | 1148 | 481 |
| C208 | 41 | 13 | 607 | 363 |
| C208 | 49 | 12 | 773 | 273 |
| C209 | 31 | 9 | 1046 | 626 |
| C209 | 62 | 21 | 1101 | 504 |
| C209 | 63 | 15 | 828 | 396 |
| C210 | 76 | 27 | 960 | 368 |
| C210 | 74 | 17 | 938 | 310 |
| C210 | 59 | 22 | 1602 | 571 |
| C211 | 46 | 17 | 708 | 352 |
| C211 | 43 | 13 | 1254 | 736 |
| C211 | 46 | 13 | 854 | 335 |
| **TOTAL** | **615** | **184** | **11819** | **5315** |

| **PAG - Context** | **Total FG** | **FG-Fos** | **Total DAPI** | **Total FOS** |
| --- | --- | --- | --- | --- |
| C212 | 40 | 32 | 762 | 191 |
| C212 | 43 | 33 | 1069 | 323 |
| C212 | 35 | 26 | 951 | 282 |
| C213 | 40 | 28 | 1150 | 305 |
| C213 | 76 | 56 | 1340 | 392 |
| C213 | 76 | 56 | 1239 | 336 |
| C214 | 60 | 48 | 1144 | 398 |
| C214 | 51 | 39 | 1125 | 291 |
| C214 | 52 | 31 | 744 | 229 |
| C215 | 53 | 40 | 1187 | 356 |
| C215 | 38 | 31 | 1045 | 245 |
| C215 | 53 | 44 | 1083 | 302 |
| **TOTAL** | **617** | **464** | **12839** | **3650** |
